# Efficient occupancy model-fitting for extensive citizen-science data

**DOI:** 10.1371/journal.pone.0174433

**Published:** 2017-03-22

**Authors:** Emily B. Dennis, Byron J. T. Morgan, Stephen N. Freeman, Martin S. Ridout, Tom M. Brereton, Richard Fox, Gary D. Powney, David B. Roy

**Affiliations:** 1 School of Mathematics, Statistics and Actuarial Science, University of Kent, Canterbury, United Kingdom; 2 Butterfly Conservation, Manor Yard, East Lulworth, Wareham, United Kingdom; 3 Centre for Ecology & Hydrology, Benson Lane, Crowmarsh Gifford, Wallingford, United Kingdom; Oxford Brookes University, UNITED KINGDOM

## Abstract

Appropriate large-scale citizen-science data present important new opportunities for biodiversity modelling, due in part to the wide spatial coverage of information. Recently proposed occupancy modelling approaches naturally incorporate random effects in order to account for annual variation in the composition of sites surveyed. In turn this leads to Bayesian analysis and model fitting, which are typically extremely time consuming. Motivated by presence-only records of occurrence from the UK Butterflies for the New Millennium data base, we present an alternative approach, in which site variation is described in a standard way through logistic regression on relevant environmental covariates. This allows efficient occupancy model-fitting using classical inference, which is easily achieved using standard computers. This is especially important when models need to be fitted each year, typically for many different species, as with British butterflies for example. Using both real and simulated data we demonstrate that the two approaches, with and without random effects, can result in similar conclusions regarding trends. There are many advantages to classical model-fitting, including the ability to compare a range of alternative models, identify appropriate covariates and assess model fit, using standard tools of maximum likelihood. In addition, modelling in terms of covariates provides opportunities for understanding the ecological processes that are in operation. We show that there is even greater potential; the classical approach allows us to construct regional indices simply, which indicate how changes in occupancy typically vary over a species’ range. In addition we are also able to construct dynamic occupancy maps, which provide a novel, modern tool for examining temporal changes in species distribution. These new developments may be applied to a wide range of taxa, and are valuable at a time of climate change. They also have the potential to motivate citizen scientists.

## Introduction

The study of species distributions is an important and expanding area of ecological research, allowing the investigation of factors affecting species occurrence, as well as analysis of changes in species’ range and distribution [1]. Often the primary sources of distribution data are opportunistic, presence-only citizen-science records [2]. These data, which are relatively unstructured, are often available in large quantity and over extensive geographic areas and time periods. They are inherently biased [3], for example with variation in coverage both spatially and temporally, and suitable methods are required to produce robust and unbiased measures of distribution change from such data [4]. Throughout this paper we shall analyse presence-only citizen-science data.

Opportunistic distribution recording schemes exist for a wide variety of taxa. In the UK, for example, the Biological Records Centre oversees recording schemes for 85 taxonomic groups, for which data are made available through the National Biodiversity Network gateway [5], which at a greater scale form part of the Global Biodiversity Information Facility (GBIF), which holds over 600 million occurrence records for 1.6 million species (http://www.gbif.org/). Covering primarily North America, but also many other countries, more than 17 million checklists for birds are collated by eBird, for which a “Big Data” approach has been described [2, 6].

When presence-absence information with replicate observations is available, occupancy models [7] are a popular choice to model distribution data [8] as they allow for imperfect detection and provide inference on a parameter denoting the probability that a site is occupied. Ignoring imperfect detection can bias estimates of occupancy [9]. For some opportunistic data non-detection records can be constructed from the sightings of other “benchmark” species [10], although within-season replication is required for at least some sites in order to separate detection probability from occupancy probability.

Biases associated with presence-only opportunistic citizen-science data which can be addressed with the aid of occupancy models are discussed in [11], namely geographical bias in the distribution of surveyed locations, observation bias via variation in observer effort, and reporting bias where observers may not record all species observed. A simulation study by [4] favoured occupancy models for estimating robust distribution trends from opportunistic data.

Occupancy models have been applied to opportunistic records of various taxa including dragonflies, butterflies and birds [10–12], as well as being used for producing indicators for priority species and pollinators [13]. However the focus is often upon spatial change within a single year or temporal change in occupancy (via time series), although [14] assessed temporal changes in the occupancy of bees in the context of neonicotinoid use.

**Fig 1:**
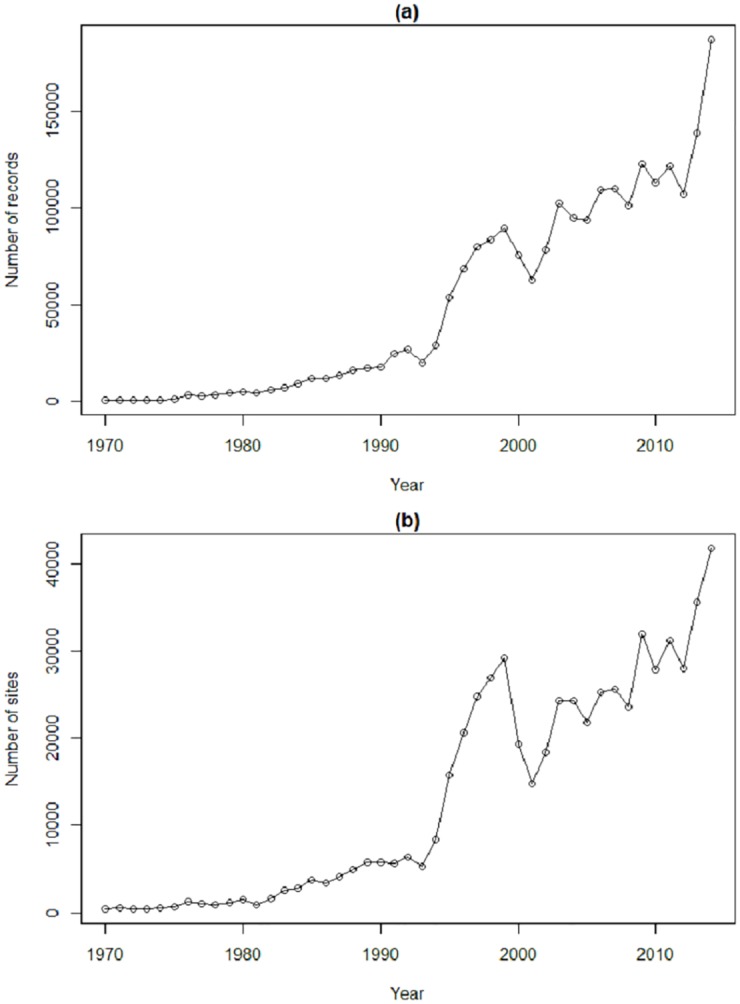
a) The number of BNM records as a function of time and b) the number of sites (1 km squares) with records each year.

We are particularly motivated by the use of occupancy models in the State of UK Butterflies 2015 report [15], to analyse data from the Butterflies for the New Millennium (BNM) recording scheme [16], and produce national indices for UK butterflies. The BNM database comprises over 11 million species occurrence records submitted mostly by volunteer members of the public, and the increase in the volume of such data over time can be seen from Fig 1.

The national report by [15] for the analysis of the BNM data was the first wide-scale application of occupancy models to UK butterflies (see also [17]), informed by [4] which employed Bayesian inference with random effects, and used list length (number of species recorded per visit) to describe variation in detection probability. This application of occupancy models uses a Bayesian approach for model fitting, which is computationally demanding and requires powerful computer clusters, resources which are often not available or feasible financially, and a limitation to the wider adoption of these models. This is especially true as models usually need to be fitted to data for multiple species. Bayesian implementations of occupancy models may typically use random effects to describe site effects on occupancy and detection probability. Because of the hierarchical framework of occupancy models, fitting them with random effects using classical inference is not straightforward, though one potential approach is outlined in [18]. Instead, we build upon the work of [17], who describe site variation in occupancy through appropriate fixed covariates in a standard manner, and we shall also model variation in detection through fixed covariates, which is more tractable within a classical framework, and can potentially be used to identify important ecological factors. Additionally, by analysing data separately for each year, model fitting is simplified and annual updates can be created from only the most recent data.

The work of this paper has more general application and relevance, particularly to large-scale multi-species analysis of opportunistic occurrence records; for example the State of Nature report for the UK applies occupancy models to 1,589 terrestrial and freshwater species [19], following the Bayesian implementation of [4].

We model the occurrence of UK butterflies using efficient methods of classical statistical inference, drawing comparisons with the results of a Bayesian implementation in [15]. We present the standard occupancy model used and the Bayesian alternative from [15] and [4]. We describe the calculation of occupancy indices, and present both the illustrative butterfly species selected in the paper and the covariates included in the models. Comparisons between Bayesian and classical modelling using real and simulated data are provided, demonstrating the major efficiency gain from using classical analysis. Focusing on classical analysis, we provide new regional occupancy indices and large-scale occupancy maps, together with associated standard error displays, and introduce dynamic maps. The paper ends with general discussion and avenues for further work.

## Materials and methods

### Occupancy models

For any species, records are made at *S* sites, each surveyed *T* times within a fixed season, resulting in an encounter history **y**_*i*_ = {*y*_*i*,*j*_; *j* = 1, 2, …, *T*} for the *i*th site, where *y*_*i*,*j*_ = 1 indicates that the species was detected and *y*_*i*,*j*_ = 0 otherwise. The encounter history probability for the *i*th site is given as

$$\begin{array}{c} \text{Pr}\left(y_{i}|z_{i}=1\right)=\prod_{j=1}^{T}p_{i,j}^{y_{i,j}}{\left(1-p_{i,j}\right)}^{1-y_{i,j}},\;\text{for}\;i=1,\cdots ,S, \end{array}$$

where *z*_*i*_ is an indicator for whether the *i*th site is occupied taking the values 0/1, such that **y**_*i*_ = **0** with probability 1 when *z*_*i*_ = 0 (site *i* unoccupied), and *p*_*i*,*j*_ represents the detection probability for site *i* and visit *j*. The likelihood is then the product of all such probabilities over the set of *S* sites,

$$\begin{array}{c} L\left(\psi ,p;\left\{y_{i}\right\}\right)=\prod_{i=1}^{S}\left\{\text{Pr}\left(y_{i}|z_{i}=1\right)\psi_{i}+I\left(y_{i}\right)\left(1-\psi_{i}\right)\right\}, \end{array}$$

where *ψ*_*i*_ = Pr(*z*_*i*_ = 1) represents the occupancy probability. The corresponding probability that the site is unoccupied is (1 − *ψ*_*i*_). Detection probability can vary with site-specific covariates, as well as covariates that vary within the season. The likelihood is zero-inflated to account for the sampling of potentially unoccupied sites. Hence *I*(**y**_*i*_) denotes an indicator function which is satisfied if and only if the encounter history for the *i*th site is entirely zero, i.e. *I*(**y**_*i*_ = **0**) = 1; *I*(**y**_*i*_ ≠ **0**) = 0. For classical inference we form maximum-likelihood estimates of parameters and describe *ψ*_*i*_ by a function of *M* site-specific covariates, *w*_*i*,*m*_, where we assume $\text{logit}\left(\psi_{i}\right)=\beta_{0}+\sum_{m=1}^{M}\beta_{m}w_{i,m}$. The model fitted in the classical analyses we call model C.

The model fitted in the Bayesian analysis we call model B [4, 15]. For this case we index *ψ*_*i*_ and *p*_*i*,*j*_ with respect to year, so that

$$\begin{array}{c} \text{logit}\left(\psi_{i,t}\right)=b_{t}+u_{i}, \end{array}$$

where *b*_*t*_ is a fixed year effect for year *t* and *u*_*i*_ is a random site effect, where *u*_*i*_ ∼ *N*(0, *σ*_*u*_). The detection probability *p*_*i*,*t*,*j*_ is described by

$$\begin{array}{c} \text{logit}\left(p_{i,t,j}\right)=a_{t}+k\text{log}\left({\text{G}}_{i,t,j}\right), \end{array}$$

where *k* is a constant, G_*i*,*t*,*j*_ is the list length (number of species recorded) at site *i* in year *t* on visit *j*, and *a*_*t*_ is a random year effect, where *a*_*t*_ ∼ *N*(*μ*, *σ*). This model forms a component of the Sparta [4] package in R [20]. We assume the following prior distributions: *b*_*t*_ ∼ *U*(−10, 10); *σ*_*u*_ ∼ *U*(0, 5); *μ* ∼ *N*(0, 10); *σ* ∼ *U*(0, 5); *k* ∼ *U*(−10, 10).

The indicator variable *z*_*i*_ is also indexed with respect to *t*, so that for site *i* and year *t*, the indicator variable *z*_*i*,*t*_ is estimated from the Markov chain Monte Carlo (MCMC) used in model fitting. The annual proportion of sites occupied is then estimated and forms the index of occurrence as follows

$$\begin{array}{c} I_{t,B}=\frac{1}{n_{t}}\sum_{i=1}^{n_{t}}z_{i,t}, \end{array}$$

where *n*_*t*_ is the number of sites. Trends in occupancy were estimated from the posterior mean percentage change in fitted occupancy between 2005 and 2014.

### Model differences

There are several key differences between models B and C. The Bayesian approach necessarily uses data from all years at once because of the structure of model B. In contrast the classical approach analyses the data from each year separately, because of the structure of model C. Model B does not assume time variation in the random site effect distributions, so that occupancy is only assumed to change with time through the year effects. By contrast, in model C, year-to-year changes occur both from the occupancy intercept for each year (year effects), as well as due to temporal changes in covariates. In addition, as the covariates used in model C are indexed by site location, it is possible to use model C to estimate occupancy at a variety of levels, incorporating prediction, without any further model fitting. This is not true of model B without modification. In this paper we draw comparisons with the results and approach of [15]. In theory a Bayesian approach could be taken for single years, or incorporate covariates, but these are more tractable using classical inference, particularly, for example, for selection of covariates.

### Indexing occupancy

Suppose that in year *t*, a region of interest for any species contains *n*_*t*_ sites, with occupancy estimate ${\hat{\psi }}_{i,t}$ for site *i*. For model C, to index occupancy we simply take the average occupancy estimate in the region of interest. Thus the occupancy index *I*_*t*,*C*_ for that region in year *t* is given by

$$\begin{array}{c} I_{t,C}=\frac{1}{n_{t}}\sum_{i=1}^{n_{t}}{\hat{\psi }}_{i,t}. \end{array}$$

A weighted mean of the estimated occupancy probabilities is described in [17], however it was found to produce unreliable results, due to certain variances being estimated with poor precision.

Estimates of uncertainty for *I*_*t*,*C*_ are obtained using an efficient parametric bootstrapping approach [21, p192] [22]. We generate 1000 bootstrap resamples from a multivariate Normal distribution based on the parameters and variance-covariance matrix estimated by the fitted occupancy model. The occupancy index is then estimated for each replicate and quantiles taken to estimate 95% confidence intervals. A comparison of parametric and nonparametric bootstrap approaches is given in S1 Appendix.

For the classical analysis, occupancy trends over time were estimated by fitting a weighted linear regression to the index, with the inverse standard deviations of the bootstrap replicates for each year as weights. Associated 95% confidence intervals were derived by estimating a trend for each replicate index from the parametric bootstrap and obtaining appropriate quantiles.

In order to form regional indices, [17] found it preferable to define the points within each region of interest by taking all sites within a region, rather than taking only those at which observations of at least one species had been made in the given year. However, at the national scale this could involve too much extrapolation, and for suitable comparison we have adopted the approach in [15], taking all sites for which at least one record has been made during the time period of the study (shown in Fig A in S1 File). We now define sites for the butterfly application.

### Application to BNM data

We compare models B and C for estimating occupancy for ten representative butterfly species, which are listed in Table A in S1 File, for 1976-2014. We then present more detailed results for three of these species.

We take 1 km squares of the UK national grid as sites, as in [15]. Records from the BNM data with a precise location (1 km^2^ or less) and exact date were extracted. Squares with at least one species recorded in fewer than 3 years were excluded, as in [15]. We assume that different records in the same sample unit do not refer to different locations that vary greatly. A total of 69,936 1 km squares with records were considered.

In [15], the calendar year was taken as a period of temporal closure, when the occupancy status of each site does not change, however given the varying flight periods of butterflies, we restrict the data to be within the main period for butterfly flight (beginning of April to the end of September each year). A comparison with the results of [15] without this restriction is provided in Fig B in S1 File.

The observations of non-target species are used to generate non-detection records and form detection histories, {**y**_*i*_}, for each site. A visit to a given site is therefore defined by an occasion where either the target or one or more non-target species was observed. Detections of the non-target species outside the first and last month that the target species was observed (within April-September) in a given year were disregarded, in order to prevent non-detection records being created outside the target species’ flight period, when the target species is mostly likely not present as an adult and hence not detectable.

In 84% of cases fewer than five visits were made at each location within a given year, and only 0.5% had more than 50 visits. Hence to limit the size of the data arrays and aid computational efficiency, the maximum number of visits to a location per year was limited to 50 (removing non-detections, at random, in favour of detections of the target species where *T* > 50).

For classical inference, occupancy models were fitted using the unmarked [23] package in R. Occupancy maps are also created in R, and corresponding maps of estimated standard-error display the associated uncertainty, using the delta method to produce estimates on the probability scale using the deltamethod function in the msm package [24] in R, which was more efficient than estimating standard errors from unmarked. Dynamic occupancy maps, which show annual occupancy maps as a sequence, were created using Shiny [25], and associated data and R code are provided via FigShare (https://doi.org/10.6084/m9.figshare.4748278.v1). The occupancy indices and trend estimates presented for the Bayesian approach result directly from [15].

#### Three species

We present further results for three illustrative species. Large Skipper and Small White are wider-countryside species with relatively large ranges across the UK. Large Skipper has shown recent expansions in range and increases in abundance, and Small White populations are reasonably stable. The third species, Silver-washed Fritillary, is a habitat-specialist, found in woodlands and limited mostly to southern England; this species has started to show increases in range and abundance.

#### Covariates

For illustration, we select a set of general covariates to represent spatial variation in occupancy, where northing, easting, minimum February (on average the coldest winter month) temperature, and average monthly rainfall (mm, April-September) were included as covariates for occupancy with both linear and quadratic effects. The weather covariates were taken from historic weather-station data [26], available from www.metoffice.gov.uk/public/weather/climate-historic/, which were smoothed using a thin-plate spline [27], using the fields package [28] in R, to obtain weather covariates at a scale of 1 km^2^. Selected land cover variables were also included, but as linear effects only to reduce the model complexity. Percentage land cover was taken from a 1 km^2^ land cover map from 2007 [29], which can be downloaded from https://eip.ceh.ac.uk/lcm/lcmdata. These data consist of 10 aggregate land cover classes, but, as given in Table B in S1 File, we used five combined classes to minimise complexity. The same set of covariates was adopted for all species considered, for illustration. In practice covariate sets would be expected to vary with species, following covariate selection procedures. All covariates were standardised to have zero mean and unit variance.

In the analysis of [15] detection probability was modelled using a random effect and the single covariate of list length. We consider annual variation in the effect of list length, and since detection probability might additionally be expected to vary seasonally, as butterfly populations fluctuate according to their life-cycle, as a proxy for the seasonal variation in population size we also include the proportion of observations made of the species of interest each week. Correlations between these two covariates are low, ranging from -0.23 to 0.37 over the species considered.

### Simulation study

Models B and C were applied to varying scenarios for simulated occupancy data. For each of 200 simulations, data were simulated for 1000 sites, 10 years, and 10 annual visits. Occupancy was simulated to vary either according to a covariate simulated from a standard Normal distribution, or with respect to a Normal random effect with a variance of 5, both on the logit scale. The occupancy intercept varied from 0.4 to 0.6 in even increments over time on the logit scale. In model C the slope parameter for the occupancy covariate varied by even increments each year from -0.5 to 0.5. Detection probability was assumed to be constant, and we considered p = 0.15, 0.3. For each scenario we assume that data from 20% or 50% of sites are missing in each year. In total eight simulation scenarios were considered, and in each case both models B and C were applied. For model C we always fit a model where occupancy varies with a covariate, and for model B we always assume a random effect.

Model C was fitted separately to each year using unmarked in R, as in the application to BNM data. Model B was fitted using a subset of R code from the Sparta package [4]; we ran 3 chains and 10,000 iterations, with the first 20% discarded for burn-in and thinned by taking every third iteration. Detection probability was assumed to be constant, with a uniform prior U(-10, 10) on the logit scale. As previously, for model C the occupancy intercept, *b*_*t*_, was estimated for each year, also with a uniform prior U(-10, 10). The standard deviation of the random effect for occupancy, *σ*_*u*_ was given a uniform prior U(0, 25).

## Results

### Comparing models B and C

**Fig 2:**
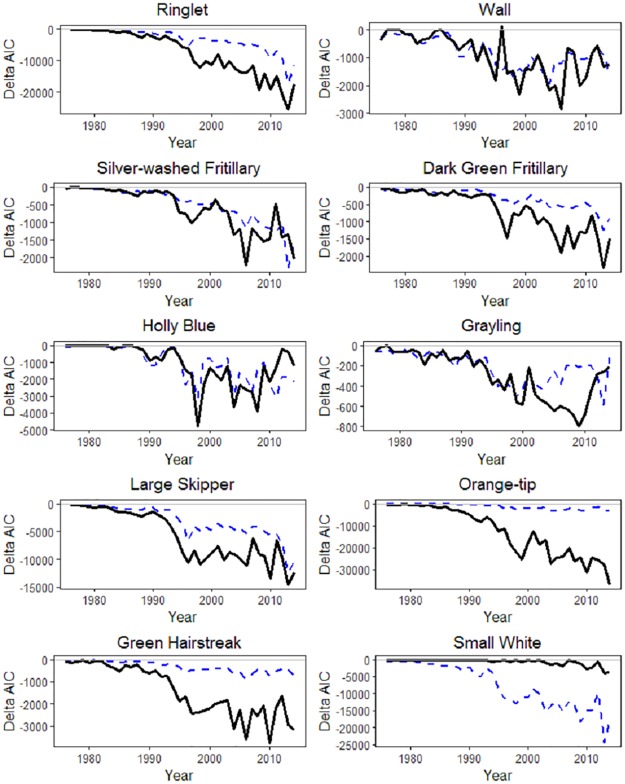
AIC comparison using varying covariates for detection probability. The solid black line compares having the seasonal covariate and list length as two covariates, rather than with just list length. The dashed blue line compares having the seasonal covariate and list length as two covariates, rather than with just the seasonal covariate.

We compare models B and C fitted to the representative set of 10 UK butterfly species, and start by considering how to model detection probability appropriately. In Fig 2 we compare the effect of using one or two covariates for detection probability using model C. The AIC differences are generally larger for later years, due to the corresponding increase in data noted in Fig 1. There is a clear conclusion that it is better to use the two covariates, rather than just one, which we do for model C.

Fig C in S1 File shows a general increase in average list length over time, particularly prior to the increase in records from 1995 ownards. Estimated annual coefficients for list length from model C (when seasonal variation is excluded) are illustrated in Fig D in S1 File. In most cases there is consistent time variation in the slope parameter, which is not a feature of model B of [15]. The estimated slopes typically vary about similar values, with the main exceptions of Orange-tip and Green Hairstreak, for which the slopes are much smaller. This may be because these two species only fly early in the year, when there are fewer butterfly species in flight, and list lengths are expected to be short. For Large Skipper, list length appears to be of increasing importance over time.

**Fig 3:**
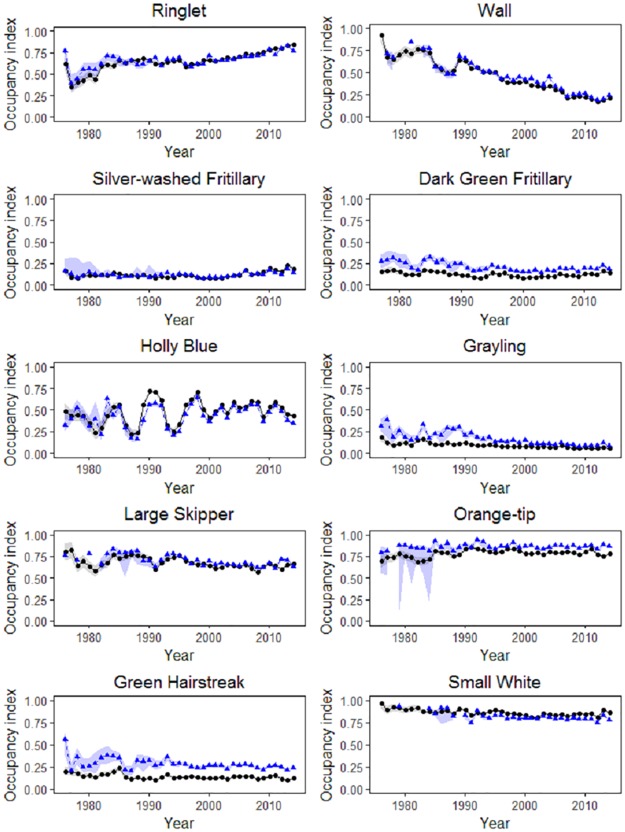
Index Comparison for models B (black, circles) and C (blue, triangles). The 95% confidence bands follow directly from the MCMC of the Bayesian analysis and from an approximate bootstrap approach in the classical case. In model C, detection probability has two covariates, the seasonal covariate and list length, and the data are restricted in date.

We now compare the indices obtained using models B and C in Fig 3. Although the agreement varies between species, in most cases the two indices show high and significant correlations at short- and long-term scales (Table C in S1 File), and we can expect even better agreement if covariates selected are matched to the characteristics of individual species. Agreement is especially good for the early years, considering the far smaller number of sites recorded then (see Fig 1). There may also be differences due to variation in how detectability was modelled, and in the exact data used, although Fig B in S1 File shows similar results when we replicate the approach in [15], except for using covariates instead of random effects, and allowing the slope for list length to vary annually.

A small number of classical estimates are not presented, which occurs for early years, when the amount of data is substantially smaller than for later years, and the model-fitting fails. This issue can be resolved by repeating the numerical optimisation used to obtain maximum-likelihood estimates from a wider range of alternative starting values for the model parameters, or by performing model selection in search of optimal covariates.

**Fig 4:**
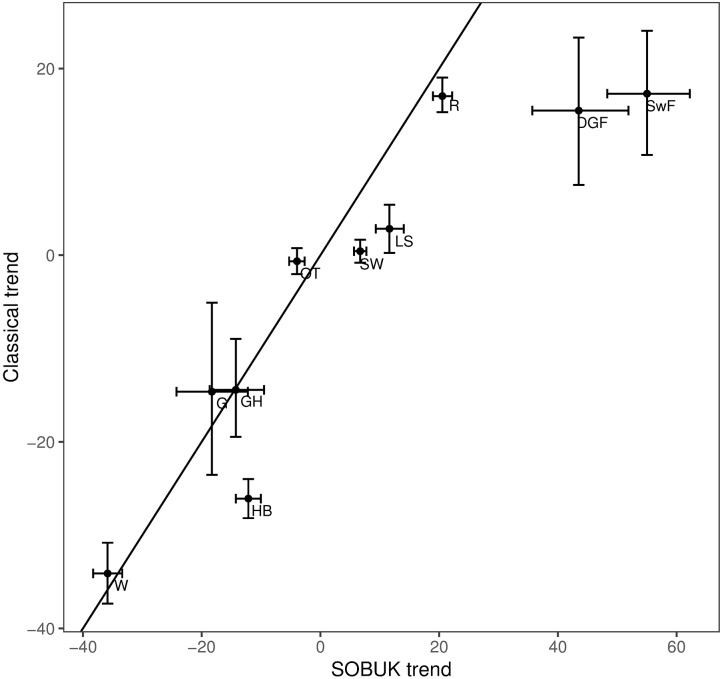
Trend comparison for models B and C. Trend comparison for 2005-2014 from model C and the trend from [15]—the State of UK Butterflies 2015 (SOBUK). Species codes are defined in Table A in S1 File. The 95% confidence bars for model C and result from the approximate bootstrap approach, while for model B we use the results of [15].

Fig 4 compares estimated trends from the two occupancy indices for 2005-2014, and suggests that the trends from the classical approach are estimated as slightly more negative than those from the Bayesian approach. Wider confidence intervals for some species trends are related to wider confidence intervals of the indices, particularly for earlier years in the series, relative to the size of the index values. We see also that the intervals for model B are generally shorter than those for model C, which is probably due to the fact that model B uses information from all years at all times.

A check of the performance of the two model fitting methods is provided by simulation (Table 1). It appears that both methods are working correctly. They produce similar results, in spite of using different models and different model-fitting procedures. The root-mean-square-error comparisons reflect in part the fact that model B uses the data for all years, whereas model C only uses the data for each year at a time. This is one reason why fitting model C is approximately 50 times faster than fitting model B. Another is how models with random effects can result in poor mixing when MCMC is used; see [30, p82]. In the remainder of the paper we fit model C.

**Table 1:** Simulation check of the Bayesian (B) and classical (C) models.

	M	*p*	Median		Mean		RMSE	
			B	C	B	C	B	C
a)	0.5	0.15	0.499	0.500	0.501	0.501	0.028	0.030
	0.2	0.15	0.501	0.500	0.501	0.500	0.022	0.024
	0.5	0.30	0.500	0.501	0.500	0.501	0.023	0.023
	0.2	0.30	0.499	0.499	0.500	0.500	0.018	0.018
b)	0.5	0.15	0.502	0.501	0.502	0.501	0.026	0.030
	0.2	0.15	0.501	0.500	0.501	0.501	0.021	0.024
	0.5	0.30	0.499	0.501	0.500	0.500	0.021	0.023
	0.2	0.30	0.499	0.500	0.500	0.500	0.018	0.018

The data were simulated based on a) a covariate and b) a random effect, for occupancy. The true median and mean occupancy estimates were both 0.5 for all scenarios. M represents the proportions of sites missed per year and *p* the detection probability. RMSE denotes the root-mean-square-error.

### Further analyses from the classical analysis

For illustration, Table 2 presents the estimated regression coefficients for model C for five years for Large Skipper. In practice a covariate selection procedure would be necessary for each species separately. For Large Skipper, in most years the weather covariates were important. Land cover covariates might be expected to have similar effects over multiple years, which is largely true for these five years for the significant covariates. Coefficients for woodland and mountain seem to be significant and positive, whereas there is a negative relationship with urban land cover. Both grassland and arable might be omitted, but this would require further investigation.

**Table 2:** Estimated covariate coefficients for occupancy for Large Skipper.

Parameter	2010	2011	2012	2013	2014
Intercept	1.751**	3.176**	2.199**	4.174**	3.211**
North	0.723	0.974**	−0.209*	−1.024**	−0.507
East	0.076	1.072**	0.949**	1.903**	0.216
North^2^	−0.922**	−1.005**	−0.545**	−0.482**	−1.503**
East^2^	−0.354**	−0.362**	−0.541**	0.162	−0.004
Temp	0.184	1.404*	1.092**	−0.366	−3.241**
Temp^2^	0.734**	0.623**	0.594**	0.157**	2.021**
Rain	−0.386	1.974**	0.693**	2.075**	−1.548**
Rain^2^	−0.007	−0.943**	0.295**	−0.976**	−0.921**
Woodland	0.57**	0.723**	0.708**	0.634**	0.544**
Grassland	−0.125	0.085	0.108	−0.126	−0.186*
Arable	−0.115	0.134	0.233**	−0.035	−0.246**
Urban	−0.348**	−0.198**	−0.2**	−0.325**	−0.401**
Mountain	0.742*	0.566*	0.583**	0.953**	1.858**

5% significance is indicated by * and 1% significance is indicated by **.

**Fig 5:**
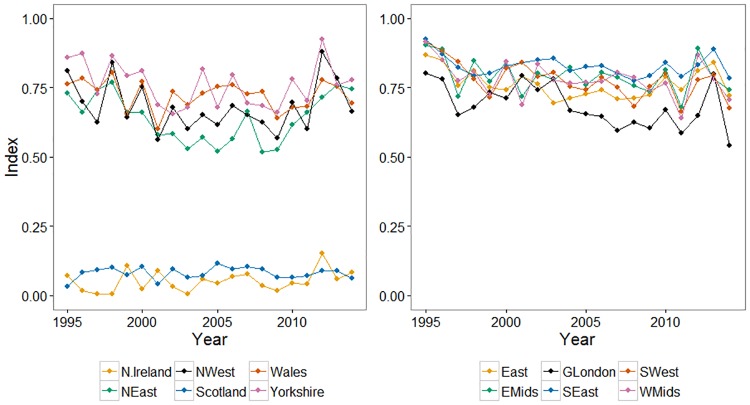
Illustrative regional occupancy indices for Large Skipper. Confidence intervals are suppressed for clarity. The indices are presented across two plots for clarity, and are loosely grouped for northern and southern regions.

We demonstrate the utility of model C for obtaining regional indices by using the geographical regions illustrated in Fig E in S1 File. We can see from Fig 5 the importance of regional indices, to complement overall national pictures.

**Fig 6:**
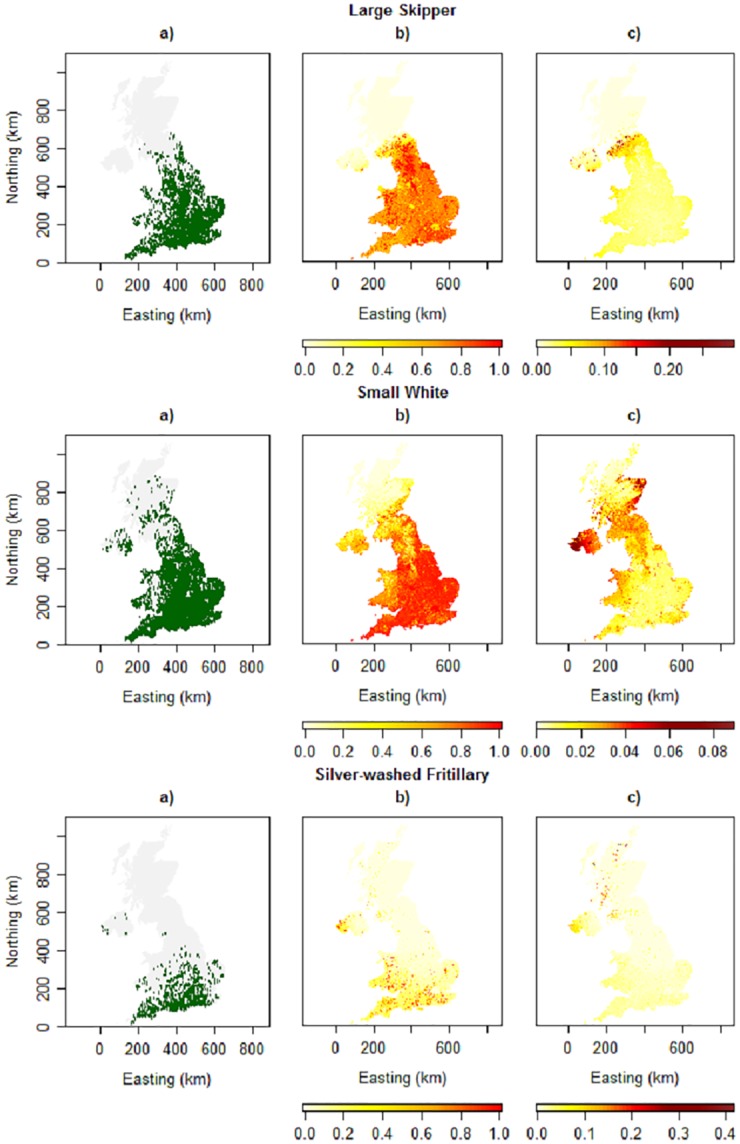
Illustrative spatial distribution maps. Maps are shown for Large Skipper, Small White and Silver-washed Fritillary in 2014: a) observations b) estimated occupancy probability c) estimated standard error.

Fig 6 presents occupancy maps for 2014 for three species. Despite a lack of appropriate model selection for covariates, the three maps show sensible predictions, and the associated standard error maps display higher uncertainty for certain areas, for example at the northern range limit for Large Skipper. Dynamic maps which display occupancy across multiple years are provided at https://ebdennis.shinyapps.io/DynMap/.

## Discussion

This paper has shown that comparable occupancy estimates may be obtained using a model with covariates fitted by classical inference compared to a model with random effects fitting using MCMC. The primary benefits of using model C are computational efficiency and implementation of covariates. These findings have importance for the application of occupancy models to multiple sets of potentially large and long-running opportunistic data sets, which may be of particular relevance to practitioners and organisations limited in access to and funds for powerful computational resources. An efficient approach also provides the flexibility for many scientific hypotheses to be investigated, as demonstrated in this paper, for example to visualise and assess spatial as well as temporal variation and changes in occupancy.

Using random effects is a popular and useful approach in many applications, but modelling variation directly via covariates, where available, may be more informative ecologically. Model B can be modified by the addition of covariates, but model fitting will still be time-consuming. Also for fitting model C by classical inference, methods of model selection and goodness-of-fit are generally better established and suitable priors do not require selection and comparison.

Dynamic maps provide an up-to-date tool for visualising and monitoring changes in a species’ distribution, which can motivate and retain the citizen scientists that contribute data. Maps of species’ distributions are common, for example maps of observations or occurrence are available online for eBird data, but they are frequently presented for only a single year at a time. Furthermore, [31] highlighted the importance of providing associated error maps stating that “quantifying and honestly communicating the uncertainty in species distribution maps is a greatly under-appreciated but very important issue”, although of course the standard errors themselves are only estimates. Regional indices allow for the study of occupancy trends in regions of particular interest and how changes in occupancy might vary spatially over a species’ range, without having to fit models for each region. Composite indices for groups of species may be derived as in [13, 15]. Using model C, this could easily be done on a regional basis or for all squares to create maps of composite occurrence. Alternatively species’ richness may be estimated by summing species’ occupancy estimates [32, p256].

Illustrative examples have been presented in this paper, but the covariates chosen for occupancy were selected for demonstration only. Interaction terms, for example, were not considered and may be important, and alternative non-linear relationships could be accommodated [33, 34]. Only aggregated land cover classes were considered here, whereas specific subclasses may be important, particularly for habitat specialist species, for example those restricted to or favouring chalk and limestone grassland. Alternatively variables linked to species’ host plants have been shown to relate to butterfly distributions [35]. The possible omission of grassland that was found for Large Skipper—a species which lays its eggs on various grasses—could be a consequence of the covariate being a composite of different grassland types. An interesting question is whether covariate selection should be done for each year separately. In some cases that might not be necessary, as for example the effect of land cover might be supposed not to change with time. Ultimately in practice compromise is required between appropriately modelling individual species’ occupancy whilst minimising complexity. Goodness-of-fit also requires consideration, for example possibly using receiver operator characteristic (ROC) curves to assess model performance [17].

It is important that the probability of detection is well modelled in order to avoid biases in the estimation of occupancy. We allowed detection probability to vary with a species’ seasonal variation in abundance by using the proportion of observations made per week, and found this to be an important covariate which was not accounted for in [15]. A similar metric was used for predicting phenology by [36], although a spline may also provide a suitable approach [37]. Other applications to taxa such as butterflies have used date as a covariate for detection probability, but have limited the analysis to a single brood for bi- or multi-voltine species [11]. Occupancy models that model arrival and departure to estimate phenology have also been developed [38, 39].

Occupancy indices could be produced for alternative regions or areas to those demonstrated here, for example for particular land cover types, urban areas, climatic regions [40], or specific areas or sites of interest, or alternatively using a clustering mechanism. The paper of [22] displayed changes in the abundance of farmland birds for each 100 km square in the UK on a map and regional occupancy indices could be visualised in a similar way. Other similarities with the study by [22] may be drawn, where biodiversity is predicted spatio-temporally. An approximate parametric bootstrap is also beneficially used in that case.

The choice of benchmark species could be fine tuned [17], for example a regional approach could be adopted, since in Scotland the expected list length will vary considerably compared to southern England. The paper of [41] adopted a regional benchmarking approach for analysing the occurrence of bryophytes, but in that case species richness was much higher than in this paper.

The models of this paper do not include spatial autocorrelation. There may be benefit in accounting for spatial autocorrelation in occupancy probability [42], for example [43] explicitly account for relative distances between sites as well as the influence of local density on occupancy and temporal dependencies. However these approaches may be computationally draining for multi-species, multi-year analyses, particularly at fine spatial scales over potentially large ranges. An exploratory look at estimates of Moran’s I, using R [44], for the covariates considered here suggested relatively low spatial autocorrelation. In order to provide a check we estimated Moran’s I for the residuals from model C, using the approach of [45], for samples of species and years. There was little indication that the models need to include spatial autocorrelation, although an exhaustive study has not been undertaken. We note also the caution of [42] regarding the dangers of naively including spatial autocorrelation when analysing large data sets.

Combining multiple sources of information has been suggested by [46]. [47] presents a Bayesian hierarchical model that describes temporal variation in range size and abundance by combining BNM data with monitoring scheme data at the 10 km scale, but discusses the potential limitations for widespread application.

Dynamic occupancy models [32] estimate temporal changes in occupancy via relevant extinction and colonisation probability parameters. However, as indicated by [11], applying dynamic occupancy models to large data sets can be computationally intensive, particularly in a Bayesian framework. Fitting simple occupancy models to data for each year separately is computationally efficient, and does not necessarily require the models to be fitted to all data as new records arise each year. A multi-year approach may nevertheless be beneficial for less well-studied taxa for which data may be poor in some years. For the classical approach, fixing covariate effects across years may be favourable in this scenario.

Opportunistic schemes are commonly used to form atlases for various taxa around the world and the methods of this paper are likely to be applicable to other species groups. In the UK alone, 85 recording schemes exist for mapping the distributions of many plants and animals, and global schemes such as eBird and GBIF hold immense quantities of citizen-science data, for which we require optimal and efficient modelling approaches, to aid monitoring and understanding of changes.

## Supporting information

S1 File Supplementary tables and figures. (PDF)

S1 Appendix Comparing a parametric and non-parametric bootstrap approach. (PDF)
