# Supplementary material for: Efficient occupancy model-fitting for extensive citizen-science data
Source: PLoS One. 2017 Mar 22;12(3):e0174433. doi: 10.1371/journal.pone.0174433 (PMC5362231; doi:10.1371/journal.pone.0174433)
Supplement: S1 File — (PDF) [file pone.0174433.s001.pdf]

Supporting information:
Efficient occupancy model-fitting for extensive citizen-science data

S1 Supplementary tables and figures

Table A: Sample of ten species used in the analysis.

Common name	Latin name	Species code
Ringlet	*Aphantopus hyperantus*	R
Wall	*Lasiommata megera*	W
Silver-washed Fritillary	*Argynnis paphia*	SwF
Dark Green Fritillary	*Argynnis aglaja*	DGF
Holly Blue	*Celastrina argiolus*	HB
Grayling	*Hipparchia semele*	G
Large Skipper	*Ochlodes sylvanus*	LS
Orange-tip	*Anthocharis cardamines*	OT
Green Hairstreak	*Callophrys rubi*	GH
Small White	*Pieris rapae*	SW

Table B: Land cover classes for UK land cover data for 2007 from Morton et al. (2014), where the five combined classes are those used in this study.

Land cover class	Combined class
Broadleaf woodland	Woodland
Coniferous woodland	
Arable	Arable
Improved grassland	Grassland
Semi-natural grassland	
Mountain, heath and bog	Mountain
Saltwater	-
Freshwater	-
Coastal	-
Built-up areas and gardens	Urban

Table C: Pearson's product moment correlation coefficients (in ascending order) between the occupancy indices from models B and C for 1976-2014 and 1995-2014, show in Figure 3 of the paper. Significant correlations are indicated by * ($p < 0.05$) and ** ($p < 0.01$).

Species	1976-		1995-	
Green Hairstreak	0.59	**	0.67	**
Orange-tip	0.61	**	0.33	
Small White	0.73	**	0.72	**
Large Skipper	0.73	**	0.56	*
Silver-washed Fritillary	0.75	**	0.94	**
Grayling	0.80	**	0.78	**
Dark Green Fritillary	0.85	**	0.85	**
Holly Blue	0.85	**	0.98	**
Ringlet	0.87	**	0.90	**
Wall	0.98	**	0.96	**

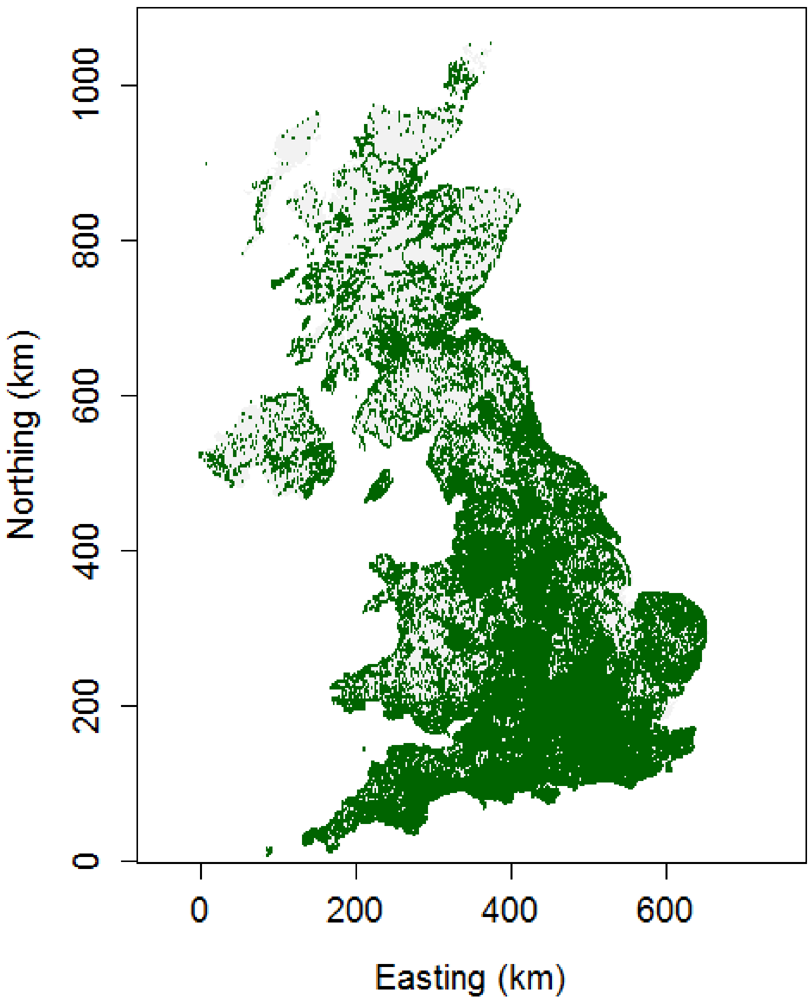

Figure A: All 1 km squares with a BNM record for 1976-2014.

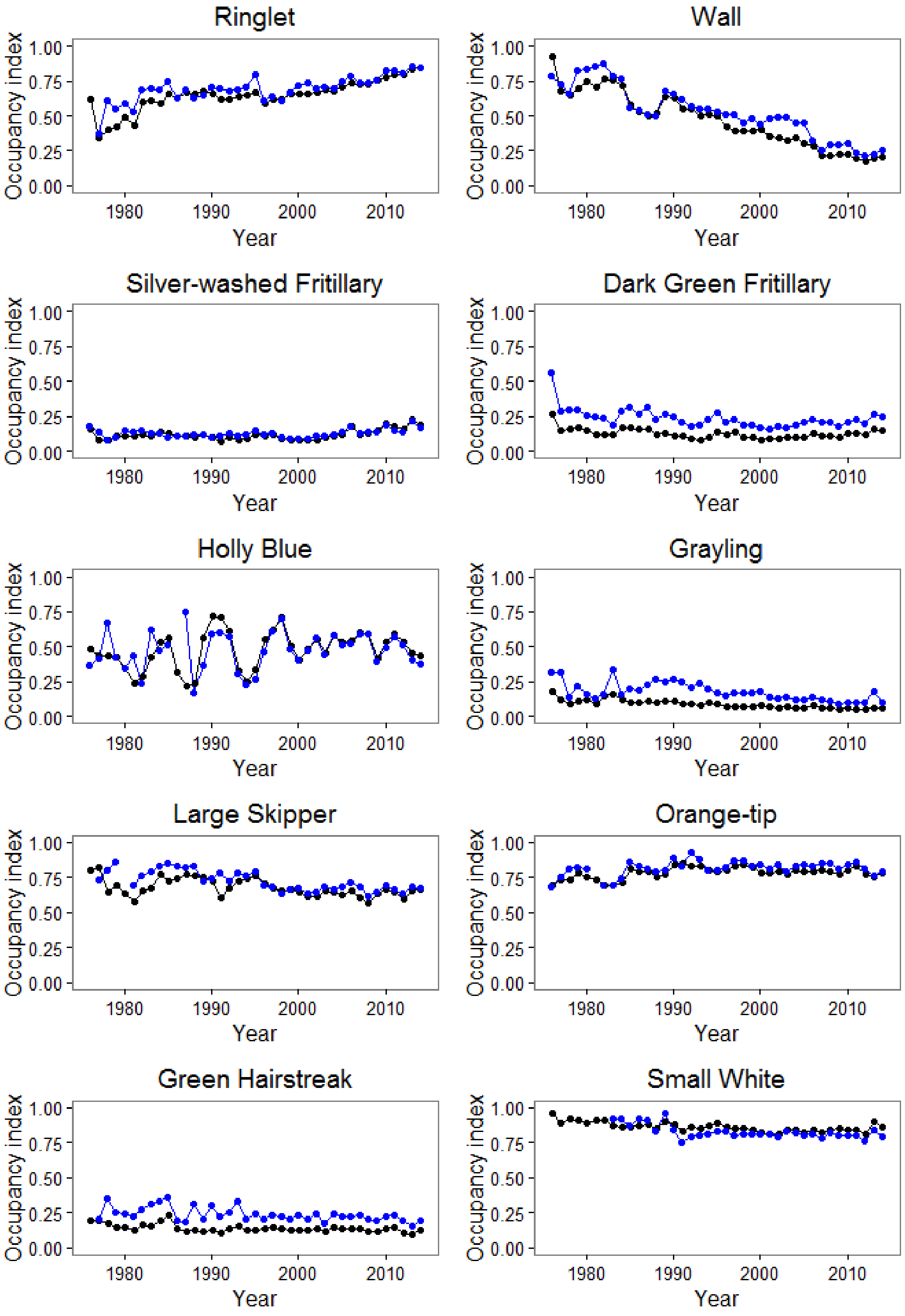

Figure B: Index comparison for models B (black) and C (blue), where the results from model C follow Fox et al. (2015), where detection probability is a function of only list length, and the data are not restricted in date. The indices from model B are identical to those in Figure 3 of the paper. There is little difference between the two analyses for recent years.

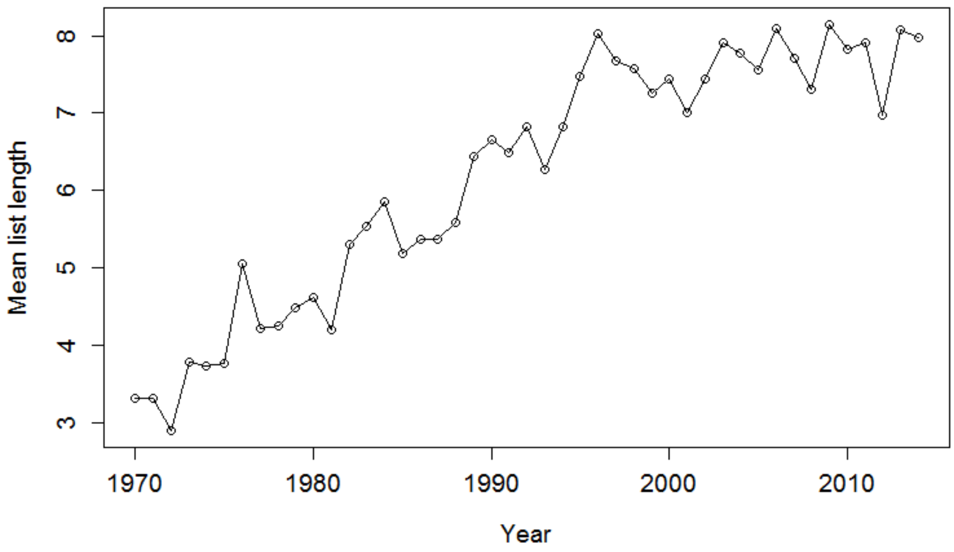

Figure C: Mean list length (across 1 km squares) per year.

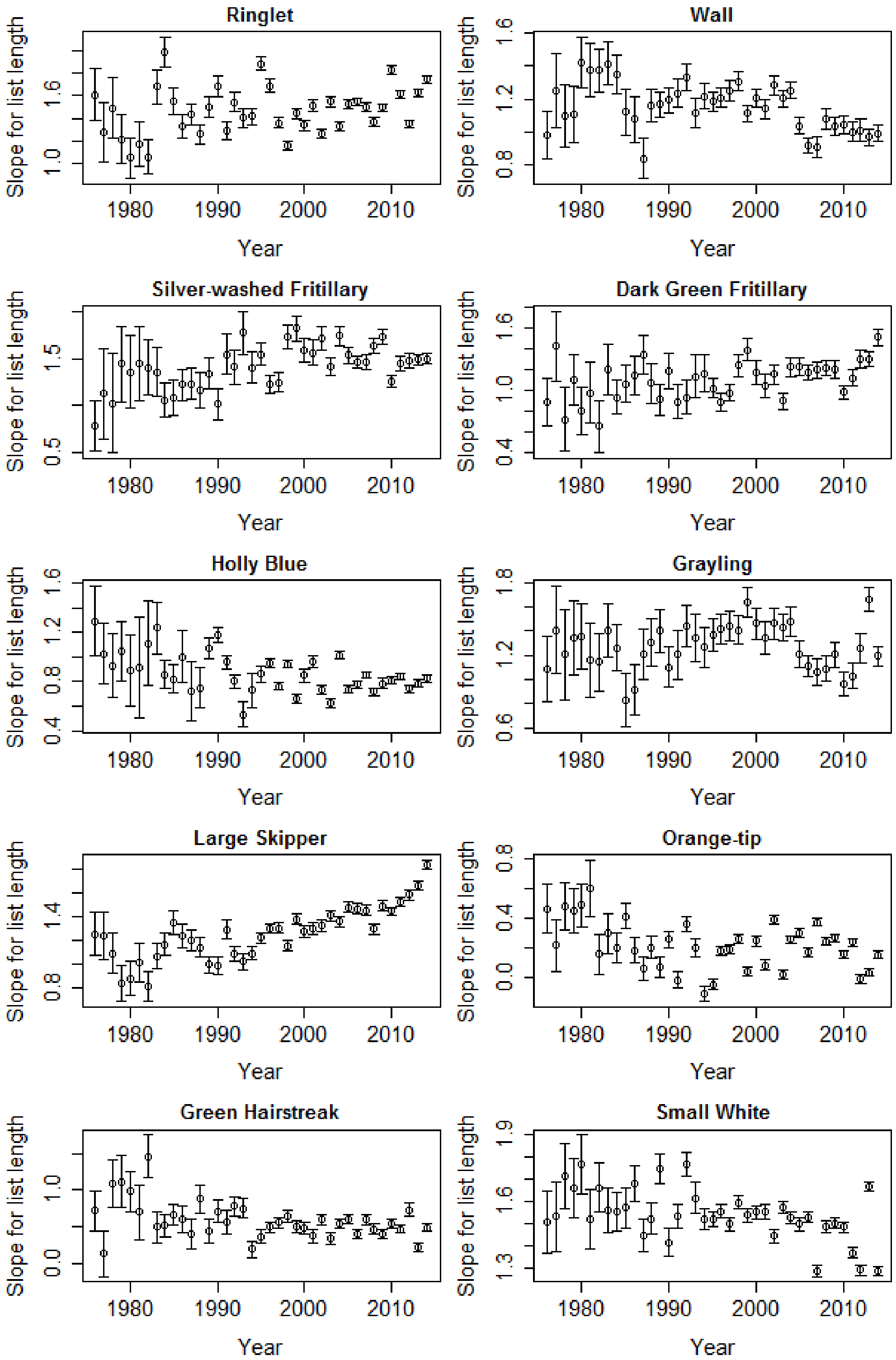

Figure D: Estimated coefficients for list length for each year and species from model C, where detection probability was considered to vary only with list length.

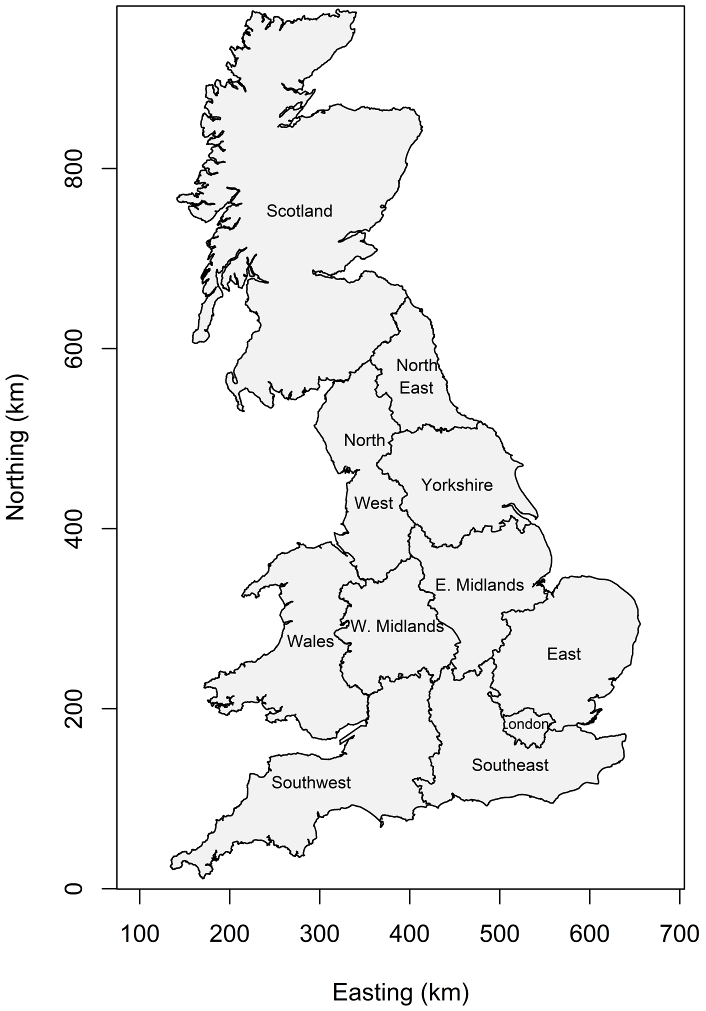

Figure E: Regions used for the computation of regional occupancy indices.
