# Supplementary material for: Efficient occupancy model-fitting for extensive citizen-science data
Source: PLoS One. 2017 Mar 22;12(3):e0174433. doi: 10.1371/journal.pone.0174433 (PMC5362231; doi:10.1371/journal.pone.0174433)
Supplement: S1 Appendix — (PDF) [file pone.0174433.s002.pdf]

Supporting information:
Efficient occupancy model-fitting for extensive citizen-science data

S1 Comparing a parametric and non-parametric bootstrap approach

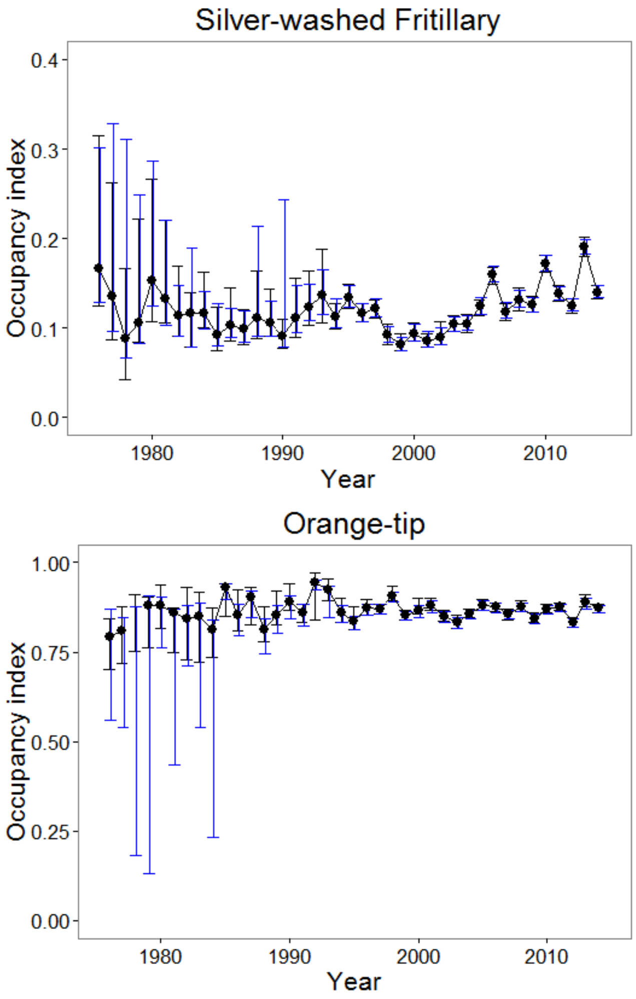

Figure A: Occupancy index from model C with confidence intervals derived from the non-parametric (black) and parametric (blue) bootstrap.

Figure A compares confidence intervals from a parametric and nonparametric bootstrap for two illustrative species. Confidence intervals estimated from the parametric bootstrap are less precise for earlier years, but produce similar estimates to the non-parametric approach

in more recent years, when more data are available. The parametric bootstrap is a factor of around 1000 times faster than the non-parametric bootstrap, based upon estimating 1000 for the parametric bootstrap and 100 samples for the nonparametric bootstrap.
